# Depletion of PHLDB2 Suppresses Epithelial–Mesenchymal Transition and Enhances Anti-Tumor Immunity in Head and Neck Squamous Cell Carcinoma

**DOI:** 10.3390/biom14020232

**Published:** 2024-02-17

**Authors:** Hongyu Li, Ziyi Wang, Huiting Liang, Xiaoyong Liu, Haichao Liu, Zehang Zhuang, Jinsong Hou

**Affiliations:** 1 Department of Oral and Maxillofacial Surgery, Hospital of Stomatology, Guanghua School of Stomatology, Sun Yat-sen University, 56 Lingyuan Road West, Guangzhou 510055, China; lihongy5@mail2.sysu.edu.cn (H.L.); wangzy39@mail2.sysu.edu.cn (Z.W.); liuxy248@mail2.sysu.edu.cn (X.L.); liuhaich@mail.sysu.edu.cn (H.L.); zhuangzh3@mail.sysu.edu.cn (Z.Z.); 2 Guangdong Provincial Key Laboratory of Stomatology, Guangzhou 510080, China; 3 Guanghua School of Stomatology, Sun Yat-sen University, Guangzhou 510055, China; 4 Department of Stomatology, The Fifth Affiliated Hospital, Sun Yat-sen University, Zhuhai 519000, China; lianght3@mail.sysu.edu.cn

**Keywords:** anti-tumor immunity, CD8^+^T, epithelial–mesenchymal transition, head and neck squamous cell carcinoma, PHLDB2

## Abstract

The role of Pleckstrin homology-like domain family B member 2 (PHLDB2) in the regulation of cell migration has been extensively studied. However, the exploration of PHLDB2 in head and neck squamous cell carcinoma (HNSCC) is still limited in terms of expression, function, and therapeutic potential. In this study, we discovered an upregulation of PHLDB2 expression in HNSCC tissues which was correlated with a negative prognosis in patients with HNSCC. Additionally, we determined that a high level of expression of PHLDB2 is crucial for maintaining cell migration through the regulation of the epithelial–mesenchymal transition (EMT). Furthermore, we demonstrated that the ablation of PHLDB2 in tumor cells inhibited tumorigenicity in a C3H syngeneic tumor-bearing mouse model. Mechanistically, PHLDB2 was found to be involved in the regulation of T cell anti-tumor immunity, primarily by enhancing the activation and infiltration of CD8^+^ T cells. In light of these findings, PHLDB2 emerges as a promising biomarker and therapeutic target for interventions in HNSCC.

## 1. Introduction

Head and neck squamous cell carcinoma (HNSCC) is a highly aggressive form of cancer that is diagnosed in approximately 800,000 individuals annually worldwide [1,2,3]. To date, various treatment modalities such as surgery, chemotherapy, radiotherapy, and targeted therapy have been developed to combat HNSCC, leading to a modest improvement in the 5-year survival rate. Despite these advances, the recurrence and metastasis of HNSCC still pose significant challenges in patient management [4,5,6]. It is therefore crucial to identify more effective therapeutic targets and enhance our understanding of their anti-tumor mechanisms in order to enhance the survival outcomes of HNSCC patients.

Immunotherapy, especially immune checkpoint inhibitors (ICIs), has achieved significant results in a wide range of tumor types and is expected to improve the prognosis of several cancers, including HNSCC [7,8]. However, the clinical efficacy of ICI is severely limited by tumor immune escape. Only 15% of patients with HNSCC are able to achieve a significant and long-lasting therapeutic response to ICIs [9,10]. Therefore, it is essential to elucidate the molecular mechanisms of tumor immune escape and develop effective therapeutic targets to improve the efficacy of immunotherapy. Previous studies have suggested a link between the infiltration of CD8^+^T cells and a lack of response to immunotherapy based on gene expression data from tumors of patients who received ICI treatments [11,12]. Thus, promoting intratumoral CD8^+^T cell infiltration may be an effective way to boost anti-tumor immunity.

The protein known as Pleckstrin homology-like domain family B member 2 (PHLDB2), which contains a PH domain, is crucial in facilitating the migration of cells by actively engaging in complex formations with various proteins including CLASPS, Prickle 1, and Liprin α1 [13,14,15], which suggests that PHLDB2 plays a significant role in the development of tumors and could potentially be a target for treating different types of cancer. Various studies have indicated that high levels of PHLDB2 expression are linked to a poor prognosis in multiple tumor types [16,17,18,19]. Furthermore, recent research has highlighted the possible involvement of PHLDB2 in cancer cell mobility and the process of the epithelial–mesenchymal transition (EMT), which ultimately leads to metastatic progression [20,21]. These findings strongly suggest that PHLDB2 may hold therapeutic potential for specific tumors. Nonetheless, the exploration of PHLDB2’s expression, function, and therapeutic significance in HNSCC remains incomplete. It has been demonstrated that the frequent activation of signaling pathways is connected to the EMT during the malignant advancement of tumors [22,23,24]. Notably, tumor cells undergoing the EMT can also produce immunosuppressive cytokines or chemokines to exacerbate the immunosuppressive state of the tumor microenvironment, contributing to tumor progression [25]. Keshamouni et al. found that a TGFβ-induced EMT with increased expression of E-cadherin, which activates the NK ligand, increases susceptibility to NK cell-mediated cytotoxicity [26]. Maehara et al. showed that vimentin, an EMT-related marker, was associated with less CD8^+^T cell infiltration and more FoxP3+ cell infiltration. Targeting the EMT signaling pathway in combination with PD-1/PD-L1 immunotherapy may improve the outcome of patients with pancreatic ductal adenocarcinoma cells [27]. Additionally, a large amount of preclinical research has shown that the inhibition of molecules linked to the EMT can successfully impede tumor progression, remodel the immune microenvironment within the tumor, and reinstate an anti-tumor immune response [25,28,29,30]. Therefore, it is crucial to investigate the potential role of PHLDB2 in the EMT and anti-tumor immunity in HNSCC.

In this study, we found that PHLDB2 was upregulated in HNSCC compared to normal tissues. A higher expression level of PHLDB2 was associated with a poorer prognosis. In terms of functionality, the genetic reduction in PHLDB2 dampened the EMT phenotype of cancer cells and boosted CD8^+^T cell function. Furthermore, we found that the depletion of PHLDB2 significantly delayed tumor growth and enhanced the infiltration and function of CD8^+^T cells in a mouse model of HNSCC bearing a C3H tumor. These results suggest that PHLDB2 is a key regulator of the anti-tumor effect and highlight the potential therapeutic value of targeting PHLDB2 in HNSCC.

## 2. Materials and Methods

### 2.1. Cell Culture

This study employed the HNSCC cell lines HN6 and CAL27, 293T cells (KCB Cat# KCB 200744YJ, RRID: CVCL_0063), and murine SCC7 cells. HN6 cells were acquired from Wayne State University, while CAL27, 293T cells, and murine SCC7 cells were procured from the American Type Culture Collection (Manassas, VA, USA). A normal epithelial cell line (NOK) was provided by the Guangdong Provincial Key Laboratory of Stomatology. For in vivo experiments, the murine SCC cell line SCC7, provided by Guangdong Provincial Key Laboratory of Stomatology, was utilized. The HNSCC cell lines, NOK, 293T cells, and SCC7 cells were cultured under the following conditions: DMEM (Gibco Carlsbad, CA, USA) supplemented with 10% FBS (Gibco [Carlsbad, CA, USA]), at a temperature of 37 °C, within a humidified incubator containing 5% carbon dioxide.

### 2.2. Real-Time Quantity PCR (RT-qPCR)

Total RNA preparation was carried out using an RNA quick purification kit (ES Science, Shanghai, China, RN001), followed by an assessment of RNA concentration using a NanoDrop one. The cDNA synthesis step was performed using a HiScript II One Step RT-PCR Kit (Vazyme, Nanjing, China, P611-01), following the instructions provided by the manufacturer. An RT-qPCR was conducted using SYBR Green master mix. Each sample was assayed in triplicate or more. The determination of relative expression levels was achieved by employing the ΔCt method and normalizing the results to GAPDH. The primer sequences for PHLDB2 and ACTB are presented below.

*PHLDB2*:

Forward primer: 5′-CCTGTTGGATGTTGAAAGCA-3′;

Reverse primer: 5′-GAGCCTGCTGAACAATGTGA-3′

*GAPDH*:

Forward primer: 5′-GTCAAGGCTGAGAACGGGAA-3′;

Reverse primer: 5′-AAATGAGCCCCAGCCTTCTC-3′

### 2.3. Western Blot

HNSCC cells were collected using a solution of 1× Cell RIPA Buffer combined with a mixture of protease inhibitors (Sigma, St. Louis, MO, USA, P7626-5g). Centrifugation at 12,000 rpm for 15 min at 4 °C separated the cell lysates. Subsequently, the denaturation of the protein extracts occurred by treating them with 6× SDS buffer, followed by boiling at 100 °C for 10 min. SDS-PAGE was employed for separation, and PVDF membranes (Roche, Mannheim, BW, Germany, No.3010040001) facilitated the transfer of the proteins. To block any potential nonspecific binding, the membranes were incubated in a solution of phosphate-buffered saline containing 3% nonfat dried milk and 0.1% Tween-20. Incubation with the primary antibodies, PHLDB2 (1:1000, ovus Biologicals), Vimentin (Cell Signaling Technology Cat# 3932, RRID: AB_2288553), E-cadherin (Cell Signaling Technology Cat# 3195, RRID: AB_2291471), and β-Tubulin (1:1000, CST, No.2148s) was conducted overnight at 4 °C. Afterward, horseradish peroxidase-conjugated secondary antibodies were applied at room temperature for 2 h. Finally, ECL prime Western blotting detection reagents (GE Healthcare Life Sciences, Uppsala, Sweden) were used for visualization purposes.

### 2.4. Immunohistology (IHC)

For the analysis of human HNSCC tumors, we opted to include a total of 55 paraffin blocks containing human HNSCC lesions. These particular samples were diagnosed both histopathologically and clinically at the Hospital of Stomatology, Sun Yat-sen University. To ensure proper processing, the samples were first immersed in formalin and then embedded into paraffin blocks. Subsequently, sections were submerged in an EDTA citrate buffer (with a pH of 6.0) and subjected to microwave-assisted antigen retrieval. After cooling to room temperature, the endogenous peroxide was blocked with 0.3% hydrogen peroxide for 15 min. Then, the sections were blocked with goat serum for 30 min to avoid nonspecific binding. The slides were incubated overnight at a temperature of 4 °C with the anti-PHLDB2 antibody (Novus Biologicals, Littleton, CO, USA). Subsequently, we incubated the slides with a secondary antibody that was conjugated to an HRP polymer at a temperature of 37 °C for 30 min. After that, we performed counterstaining with hematoxylin, dehydrated the slides, and mounted them with neutral gum. Finally, for image acquisition, we used an Olympus BX51 Research System Microscope equipped with 20× UPlanApo air objective lenses from Olympus, Tokyo, Japan.

### 2.5. sgRNA Transfection

A lentiviral vector was employed to introduce both the sgRNA for PHLDB2 and the control sgRNA. The construct containing PHLDB2 and the packaging plasmid (∆H8.2 and VSVG) were combined and co-transfected into 239 T cells via the lipo2000 transfection reagent. Subsequently, 72 h after transfection, the viral supernatants were gathered. To concentrate the viral particles, suitable measures were taken, and HNSCC cell lines supplemented with polybrene (2 μg/mL) were infected using the concentrated viral particles. Once the supernatant was removed by centrifugation, the transfected cells were cultured in DMEM supplemented with 10% FBS for further experimentation. Furthermore, the infected cells were selected using puromycin. The knockdown target sequence of PHLDB2 is provided below:

hPHLDB2 sgRNA #1: 5′-GAACGATTCCCAAAACATGA-3′;

hPHLDB2 sgRNA #2: 5′-AAGTGGATATCCACTGAGGG-3′;

mPhldb2 sgRNA #1: 5′-AAAGCCAACGGGGACTATTC-3′

### 2.6. CCK-8 Assay

To assess cell growth, we employed the CCK-8 assay. Briefly, the stably transduced samples of HN6 and CAL27 cells were seeded in 96-well plates at a density of 2×10^4^ cells per well with the addition of 100 μL of medium. The plates were subsequently placed in a humid environment at a temperature of 37 °C, with a 5% concentration of CO_2_, for different time intervals: 0, 24, 48, 72, 96, and 120 h. Subsequently, 10μL of CCK8 kit solution (Dojindo Molecular Technologies, Kyushu, Japan) was introduced into each well. After incubating for one hour at 37 °C with a 5% CO2 concentration in a humid atmosphere, the absorbance at 450 nm was measured using a microplate reader.

### 2.7. Survival Analysis

The application of TIMER 2.0 (http://timer.comp-genomics.org accessed on 12 May 2022) is commonly utilized to investigate the prognostic importance of genes in HNSCC. We assessed the prognostic implications of PHLDB2 expression for overall survival in HNSCC through the utilization of TCGA databases. HNSCC patients were divided into high- and low-expression groups based on the median expression levels of PHLDB2 for a survival analysis. The assessment of the *p*-value was performed by conducting a Kaplan–Meier survival analysis and employing the log-rank test.

### 2.8. Co-Expression Gene and Survival-Related Gene Analysis

We utilized the LinkedOmics database (http://www.linkedomics.orglogin.php accessed on 4 June 2022) to identify the co-expressed genes that showed correlations with PHLDB2 expression in the RNA-seq data obtained from HNSCC patients in the TCGA cohort. To calculate the Pearson correlation coefficient, we accessed the LinkedOmics website and generated a volcano map of the co-expressed genes. To visualize the co-expression results between PHLDB2 and immune-related genes, we applied the “Limma” package to generate a heatmap.

### 2.9. T Cell Cytotoxicity Assays

The cleavage caspase-3 assay was utilized to measure the T cell killing activity. Peripheral blood mononuclear cells (PBMCs) were used to acquire T cells by employing the Pan T Cell Isolation Kit (Miltenyi, North Rhine-Westphalia, Germany, No.130-096-535). To perform the experiments, 12-well plates were coated with Ultra-LEAF™ Purified anti-human CD3 (Biolegend, San Diego, CA, USA, No.300331) and Ultra-LEAF™ Purified anti-human CD28 (Biolegend, San Diego, CA, USA, No.302933) in PBS and incubated overnight at 4 °C. Isolated T cells were then seeded into 12-well plates and activated in 1640 medium for at least 72 h. The medium contained 10% FBS, 1× MEM Non-Essential Amino Acids (ThermoFisher, Waltham, MA, USA, No.11140050), 1 mM sodium pyruvate (ThermoFisher, Waltham, MA, USA, No.11360070), 100 U/mL penicillin, 100µg/mL streptomycin, and 100 IU/mL human IL2 (PeproTech, Rocky Hill, NJ, USA, No.200-02-50). HNSCC cells were previously transfected with PHLDB2 knockdown for 24 h. The HNSCC cells that were prepared beforehand were subsequently co-cultured alongside activated T cells at a proportion of 1:10 for a duration of 6 h at a temperature of 37 °C. Following this 6 h timeframe, the cells were gathered and subjected to staining using an anti-human CD3 antibody that was conjugated with APC. This staining process lasted for 30 min and took place in darkness on ice, after which the cells were washed with PBS. Following this, the cells were fixed and permeabilized prior to being subjected to staining using an anti-human/mouse-cleaved caspase-3 antibody that was conjugated with FITC (BD, Franklin Lakes, NJ, USA, No. 559341). Again, this staining process lasted for 30 min and took place in darkness on ice, following which the cells were washed once more with PBS. The percentages of tumor cells that displayed positive staining for cleaved caspase-3 were subsequently analyzed utilizing the Beckman CytoFLEX (Brea, CA, USA) instrument.

### 2.10. C3H Syngeneic Model

To investigate the functional role of PHLDB2 in HNSCC, we utilized a mouse cell line known as SCC7 (mouse squamous cell carcinoma, ATCC, IM-M102). Female C3H mice, six weeks old, were acquired from Vital River Laboratories (Beijing, China) with the reference ID MGI: 2160217. The assignment of animals to experimental groups was carried out randomly and without bias. For the initiation of tumor growth, subcutaneous injections of 1 × 10^6^ SCC7 cells suspended in PBS/Matrigel were administered in the right flank region. No exclusion of subjects occurred during our study. Tumor xenografts were allowed to grow until they reached an average volume of approximately 100 mm^3^. Subsequently, an experimenter, who remained blinded to the experimental cohort, measured the tumors every other day. The determination of tumor volumes involved the calculation (length × width^2^)/2. When the mice reached the predetermined endpoints, euthanasia was performed using the cervical dislocation method. Following tumor collection, the tumors were weighed, and samples underwent analysis using flow cytometry. All animal experiments were conducted in compliance with applicable guidelines and regulations and were approved by the Animal Experiment Ethics Committee at Sun Yat-Sen University.

### 2.11. Statistical Analysis

To determine the statistical significance of the data, Student’s *t*-test or an ANOVA was employed. All reported *p*-values were considered statistically significant if they were less than 0.05 and were two-tailed. Each experiment conducted in vitro was repeated a minimum of three times.

## 3. Results

### 3.1. Higher Expression of PHLDB2 Is Correlated with Poorer Prognosis for HNSCC Patients

**Figure 1:**
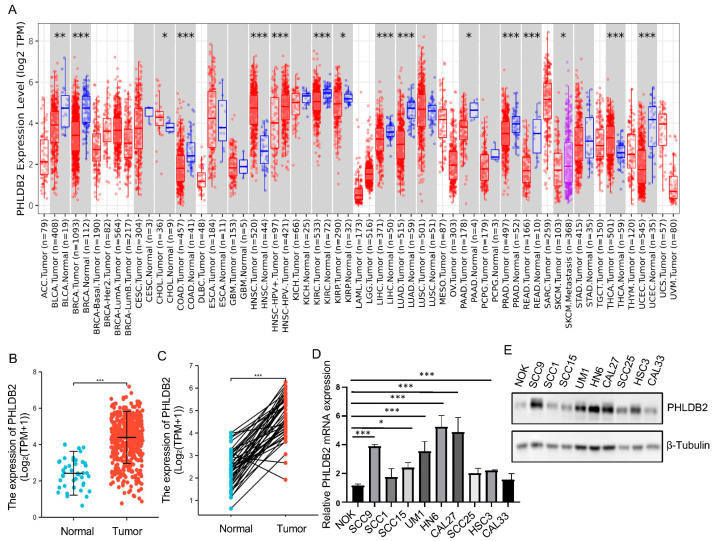

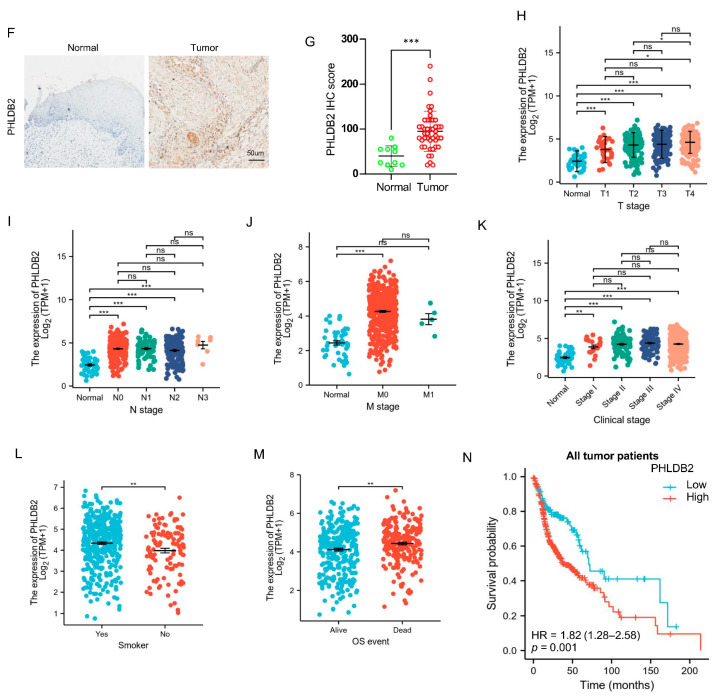
PHLDB2 is aberrantly expressed in HNSCC and associated with a poor prognosis. (**A**) TIMER was utilized to detect the expression levels of PHLDB2 in different tumors in the TCGA database. Red boxes indicated tumor tissues while blue boxes indicated normal tissues (**B**,**C**) The expression level of PHLDB2 was analyzed in normal tissues and paired adjacent tumor tissues, including unmatched tissues (**B**) and matched tissues (**C**). (**D**) Transcriptional level of PHLDB2 in different HNSCC cell lines. (**E**) The protein expression level of PHLDB2 in various HNSCC cell lines was determined through a Western blot analysis. (**F**) Representative images of PHLDB2 expression in normal tissues and HNSCC tissues via IHC staining. Scale bar: 50 μm. (**G**) PHLDB2 expression was evaluated in normal and HNSCC tissues using IHC scoring. (**H**–**M**) The expression level of PHLDB2 was analyzed in tumor tissues from patients with distinct clinical characteristics in TCGA, including T stage (**H**), N stage (**I**), clinical stage (**J**), M stage (**K**), smoker status (**L**), and OS (**M**). (**N**) The OS of TCGA patients within the HNSCC cohort was estimated using a Kaplan–Meier analysis. Data were shown as mean ± SEM values. ns means no significance. * *p* < 0.05, ** *p* < 0.01, and *** *p* < 0.001 using a two-tailed Student’s *t*-test.

To investigate the expression patterns of PHLDB2 in HNSCC, we initially assessed the mRNA expression of PHLDB2 in various types of cancers and normal tissues from different cancer populations, utilizing data from the TCGA database. Our analysis revealed a noteworthy upregulation of PHLDB2 in the majority of human cancers, including HNSCC (Figure 1A). These findings strongly indicate that the expression levels of PHLDB2 in HNSCC tumor tissues are considerably higher when compared to their corresponding normal tissues (Figure 1B). Similarly, we observed consistent results in HNSCC tissues and their paired normal tissues (Figure 1C). Additionally, we examined PHLDB2 expression in HNSCC cell lines and a normal epithelial cell line (NOK) through Western blotting and an RT-qPCR. The data showed a significant increase in PHLDB2 expression in all nine HNSCC cell lines compared to the NOK line, with the highest expression observed in the HN6 and CAL27 cell lines (Figure 1D,E and Figure S1A). For a more comprehensive evaluation of PHLDB2 expression in HNSCC, immunostaining was performed on tumor tissues and adjacent normal tissues. Interestingly, the results indicated a significantly higher expression of PHLDB2 in HNSCC compared to the adjacent normal tissues, supporting the findings obtained from the TCGA database analysis of the HNSCC cohort (Figure 1F,G).

The relationships between the expression of PHLDB2 and clinicopathological parameters, as well as patient survival, were investigated in our study. A summary of the association between PHLDB2 expression and clinical features in patients with HNSCC can be found in Table 1. Our findings revealed a significant correlation between high levels of PHLDB2 expression and various parameters including the T stage, N stage, M stage, histologic grade, clinical stage, smoker status, and overall survival (OS) of these patients (Figure 1H–M). To assess the prognostic value of PHLDB2 expression in HNSCC, we conducted a Kaplan–Meier analysis and surprisingly found that patients with elevated PHLDB2 expression levels experienced poorer outcomes (Figure 1N). A further subgroup analysis was conducted to identify specific cases in which high PHLDB2 expression was significantly linked to an unfavorable prognosis in HNSCC. These cases included male patients (HR = 1.90, *p* = 0.004), N1 (HR = 2.53, *p* = 0.041), N2 (HR = 3.70, *p* = 0.001), clinical stage II (HR = 4.51, *p* = 0.041), clinical stage IV (HR = 1.74, *p* = 0.013), histologic grade G1 (HR = 3.23, *p* = 0.036), histologic grade G3 (HR = 2.55, *p* = 0.015), and smokers (HR = 2.00, *p* = 0.001) (Figure S1). These findings collectively indicate that the overexpression of PHLDB2 is associated with a poorer prognosis in patients with HNSCC.

### 3.2. PHLDB2 Promotes EMT and Tumorigenicity of HNSCC

**Figure 2:**
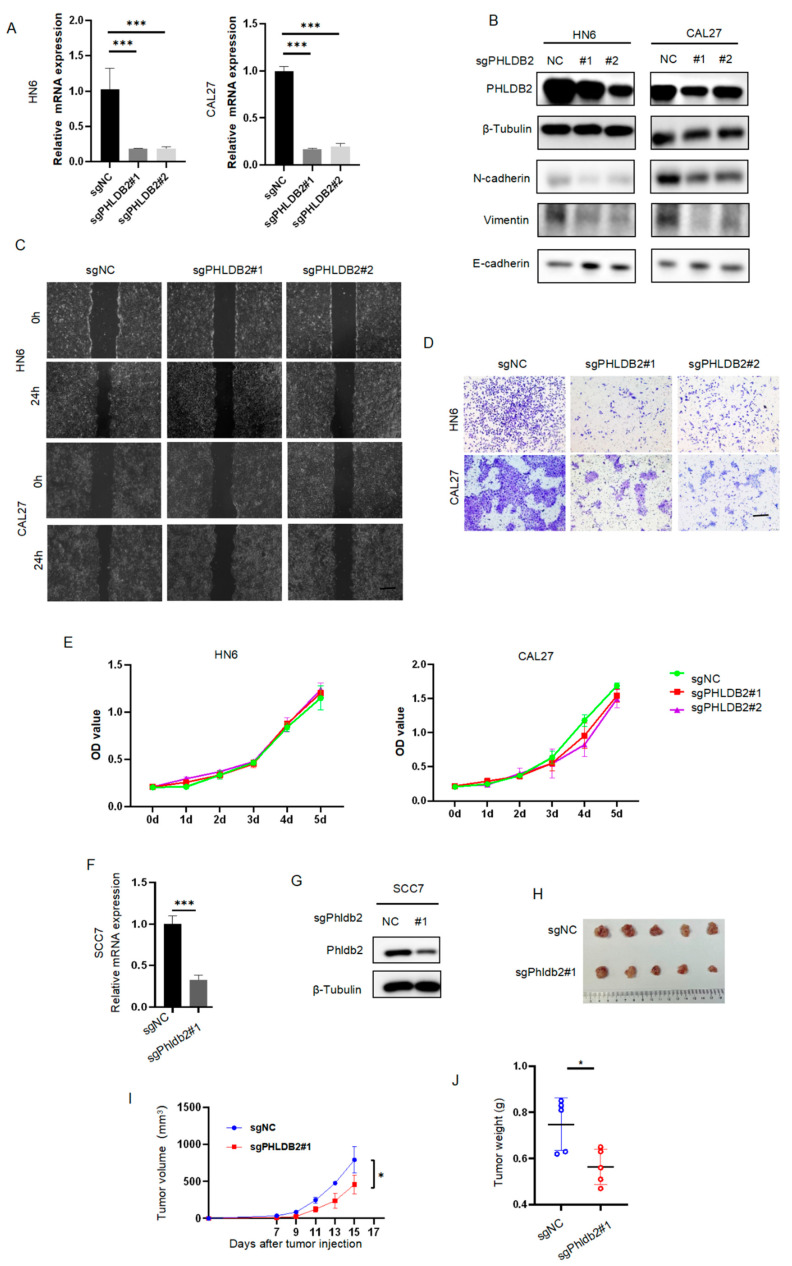
PHLDB2 promotes the EMT and the tumorigenicity of HNSCC. (**A**) The impact of reducing PHLDB2 levels was confirmed through an RT-qPCR in two HNSCC cell lines. (**B**) Western blotting was utilized to assess the expression level of an EMT-related marker in HN6 and CAL27 cells with stable PHLDB2 knockdown. (**C**) A wounding assay was performed to detect the migration ability of HN6 and CAL27 cells. (**D**) A transwell assay was performed to detect the migration ability of HN6 and CAL27 cells. (**E**) CCK-8 was used to detect the proliferation ability of HN6 and CAL27 cells. (**F**) The effectiveness of Phldb2 knockdown was confirmed through an RT-qPCR in SCC7 cell lines. (**G**) The effectiveness of Phldb2 knockdown was confirmed using Western blotting in SCC7 cell lines. (**H**) SCC7 cells stably transfected with control or Phldb2-specific sgRNAs were injected into C3H mice. (**I**) Tumor growth was monitored every 3 days; tumor size and weight were recorded. (**J**) Comparison of tumor volumes between control group and Phldb-knockdown group. Data were shown as mean ± SEM values. * *p* < 0.05, and *** *p* < 0.001 using a two-tailed Student’s *t*-test or an ANOVA.

Previous studies proposed that PHLDB2 may function as an oncogene [31,32], although its precise role in HNSCC remains poorly understood. In order to validate the functional contribution of PHLDB2 to the development and progression of HNSCC, we generated two lentivirus-based small-guide RNAs (sgRNAs; sg1 and sg2) to target two different sequences of PHLDB2. RT-qPCR and Western blot analyses were then performed to confirm the effectiveness of knockdown. The expression levels of PHLDB2 were significantly reduced after the transfection of PHLDB2 sgRNA in HN6 and CAL27 cells compared to the control cells (Figure 2A,B and Figure S2A). The results showed an inverse correlation between PHLDB2 expression and the epithelial phenotype as well as a positive correlation with the mesenchymal phenotype, which is consistent with previous reports. To examine the impact of PHLDB2 on HNSCC cell migration, we performed wound-healing and transwell assays. As shown in Figure 2C,D, PHLDB2 knockdown significantly impaired cell migration capabilities in both cell lines compared to the control group (Figure S2B,C). Furthermore, we found that the depletion of PHLDB2 did not have a significant effect on the proliferation of HN6 and CAL27 cells, as measured by the CCK-8 assay (Figure 2E). Moreover, we evaluated the effect of PHLDB2 on HNSCC tumorigenicity in vivo by subcutaneously injecting SCC7 cells into C3H mice. The results demonstrated significant reductions in tumor volume and weight in the PHLDB2-knockdown cells compared to those in control mice (Figure 2F–J). These findings suggest that silencing PHLDB2 effectively inhibits the growth of HNSCC tumors in mice.

### 3.3. Elevated PHLDB2 Is Closely Related to Immune Regulation in HNSCC

**Figure 3:**
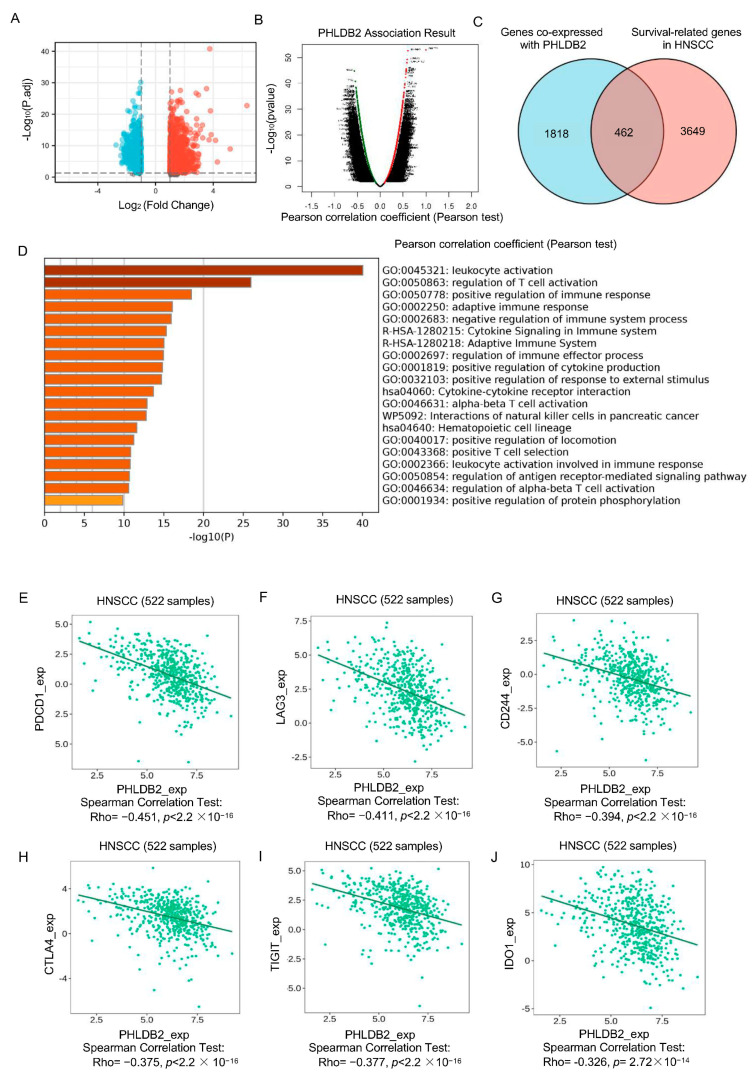
Elevated PHLDB2 is closely related to immune regulation in HNSCC. (**A**) The volcano plot exhibits the co-expression of genes with PHLDB2 in HNSCC patients from the TCGA dataset using LinkedOmics. (**B**) The volcano plot shows a total of 2280 co-expressed genes that exhibited significant correlations with PHLDB2. (**C**) A Venn diagram illustrates the genes associated with PHLDB2, as well as those genes related to HNSCC survival and those upregulated in HNSCC. (**D**) GO term analyses were performed on the genes related to PHLDB2 and the survival-related genes in HNSCC. (**E**–**J**) PHLDB2 expression is negatively correlated with the immunomodulators PDCD1 (**E**), LAG3 (**F**), CD244 (**G**), CTLA4 (**H**), TIGIT (**I**), and IDO1 (**J**) in HNSCC.

To gain a deeper understanding of the potential mechanisms underlying the promotion of HNSCC tumorigenesis by PHLDB2, we initially investigated the co-expression of genes with PHLDB2 in HNSCC patients from the TCGA dataset using LinkedOmics (Figure 3A). The findings revealed a total of 2280 co-expressed genes that exhibited significant correlations with PHLDB2 in HNSCC (FDR < 0.05, *p* < 0.05, and |cor| ≥ 0.3). Out of these 2280 genes, 927 showed a positive correlation with PHLDB2 expression, while 1354 displayed a negative correlation (Figure 3B). When comparing these 2280 significantly co-expressed genes of PHLDB2 with the 4115 prognostic-related genes mentioned earlier (using the Draw Venn diagrams online tool), we identified 462 overlapping genes for further functional analysis. This set consisted of 164 positively upregulated genes and 299 negatively downregulated genes (Figure 3C). To examine the biological functions associated with these 462 overlapping genes, we conducted GO analyses using the Metascape database. Figure 3D presents the top 20 enriched sets obtained from the enrichment analysis. The results of the analysis suggested that PHLDB2 and its associated genes serve as functional mediators in immunological modulation, specifically influencing processes such as leukocyte activation, the regulation of T cell activation, and adaptive immune response. Additionally, our study revealed that these genes are implicated in the positive regulation of cytokine production, cytokine–cytokine receptor interaction, and the regulation of the antigen receptor-mediated signaling pathway. These findings provide further evidence supporting the immunomodulatory role of PHLDB2 in the pathogenesis of HNSCC. Furthermore, we observed a negative correlation between PHLDB2 and the expression of several immune inhibitors, including PDCD1 (*r* = −0.451, *p* < 2.2 × 10^−16^), LAG3 (*r* = −0.411, *p* < 2.2 × 10^−16^), CD244 (*r* = −0.394, *p* < 2.2 × 10^−16^), CTLA4 (*r* = −0.375, *p* < 2.2 × 10^−16^), TIGIT (*r* = −0.377, *p* < 2.2 × 10^−16^), and IDO1 (*r* = −0.326, *p* = 2.72 × 10^−14^), in HNSCC (Figure 3E–J). These findings suggest that PHLDB2 maybe play a role in the regulation of T cell activation in HNSCC.

**Figure 4:**
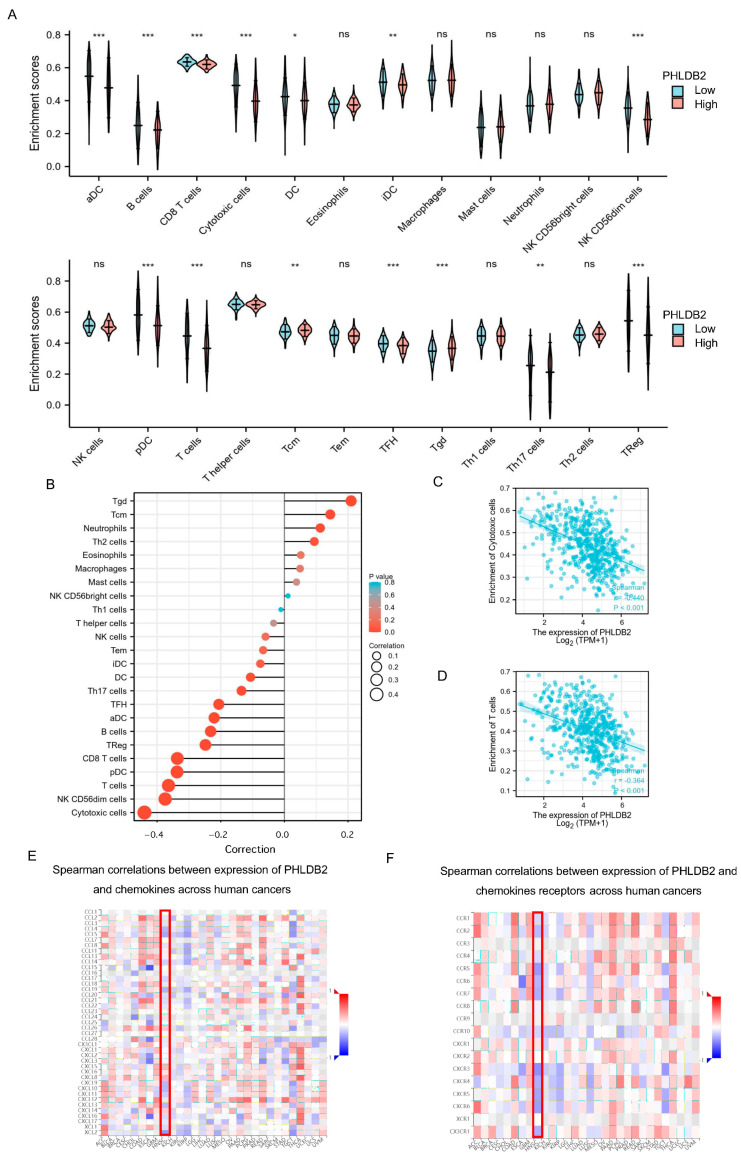
Correlation of PHLDB2 expression with immune infiltration in HNSCC. (**A**) Differential distribution of immune cells in patients with high PHLDB2 expression and low PHLDB2 expression. (**B**–**D**) Correlations between the expression level of PHLDB2 and immune cell infiltration in HNSCC (**B**), cytotoxic cells (**C**), and T cells(**D**). (**E**) Analyzing the correlation between PHLDB2 and chemokines in tumors using a heatmap. (**F**) Assessing the correlation between PHLDB2 and chemokine receptors in tumors through a heatmap analysis. ns means no significance. * *p* < 0.05, ** *p* < 0.01 and *** *p* < 0.001 using a two-tailed Student’s *t*-test.

A further analysis was conducted to examine the relationship between the expression level of PHLDB2 and the infiltration of immune cells in HNSCC (Figure 4A,B and Figure S3). Interestingly, a significant negative correlation was observed between the expression of PHLDB2 and the infiltration of multiple immune cells in HNSCC, particularly cytotoxic cells (*r* = −0.440, *p* < 0.001) and T cells (*r* = −0.364, *p* < 0.001) (Figure 4C,D). Additionally, we found that the migration of immune cells into tumors is dependent on chemokines and their receptors [33,34]. Therefore, we utilized TISIDB to investigate the relationship between the expression of PHLDB2 and chemokines, as well as their receptors, in HNSCC. The heatmap results revealed several chemokines and chemokine receptors that were negatively correlated with the expression of PHLDB2 in HNSCC (Figure 4E,F), specifically CCL4 (*r* = −0.306, *p* = 1.25 × 10^−12^), CCL5 (*r* = −0.353, *p* = 8.58 × 10^−17^), XCL2 (*r* = −0.417, *p* < 2.2 × 10^−16^), CCR5 (*r* = −0.404, *p* < 2.2 × 10^−16^), CCR6 (*r* = −0.443, *p* < 2.2 × 10^−16^), and CXCR6 (*r* = −0.385, *p* < 2.2 × 10^−16^) (Figure S4). These findings suggest a negative association between PHLDB2 and the expression of chemokines and their receptors in HNSCC. In conclusion, our data support the notion that the PHLDB2 gene may significantly impact tumor immunity.

### 3.4. Targeting PHLDB2 Exerts an Anti-Tumor Effect in HNSCC by Enhancing the Anti-Tumor Function of T Cells

**Figure 5:**
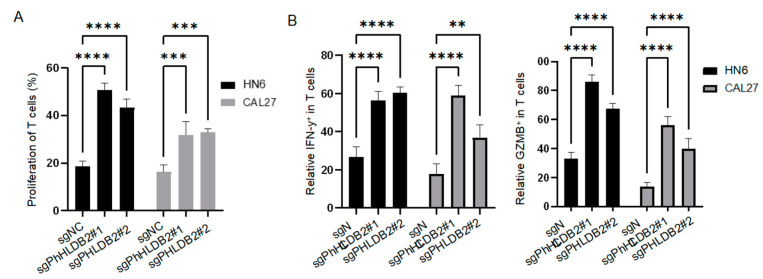

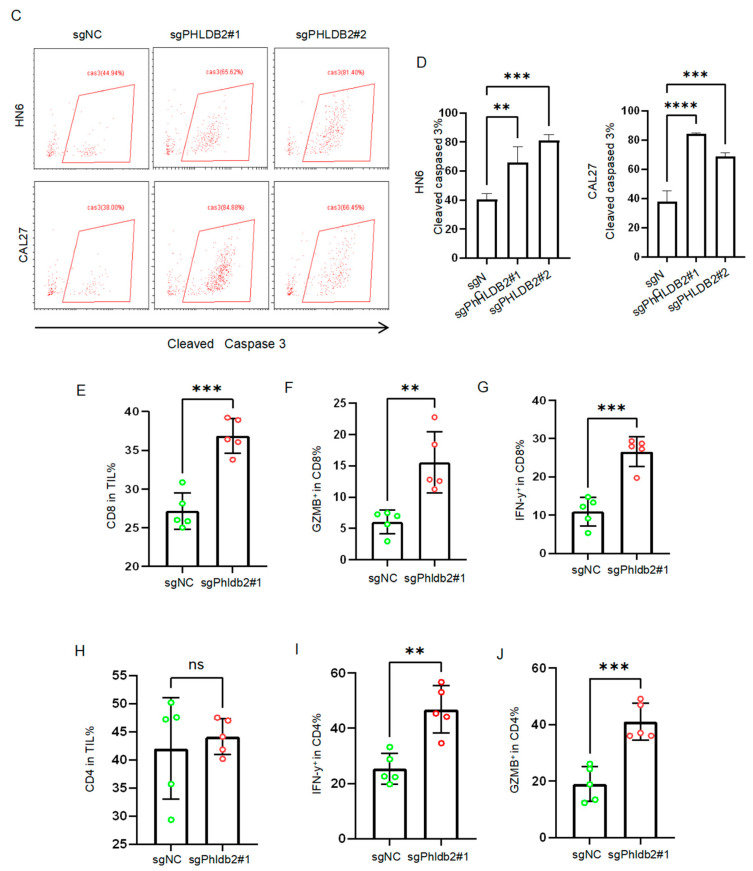
Targeting PHLDB2 exerts an anti-tumor effect in HNSCC by enhancing the anti-tumor function of T cells. (**A**,**B**) The percentages of proliferating T cells (**A**), IFNγ^+^ T, and GZMB^+^ T (**B**) were measured by flow cytometry with genetic-depletion PHLDB2 co-cultured with T cells. (**C**,**D**) Representative images (**C**) and quantification of cleaved caspase-3^+^ cells in HN6 and CAL27 cells (**D**) with genetic-depletion PHLDB2 co-cultured with T cells. (**E**) Flow cytometry showed that the percentage of CD8 in TIL was higher in the Phldb2 knockdown group compared to the control group. (**F**,**G**) Flow cytometry showed that the percentages of GZMB^+^CD8 (**F**) and IFN-γ^+^CD8 (**G**) were higher in the Phldb2 knockdown group compared to the control group. (**H**) Flow cytometry showed that the percentage of CD4 in TIL had no significant difference between the Phldb2 knockdown group and the control group. (**I**,**J**) Flow cytometry showed that the percentages of IFN-γ^+^ CD4 (**I**) and GZMB^+^ CD4 (**J**) were higher in the Phldb2 knockdown group compared to the control group. Data were shown as mean ± SEM values. ns means no significance. ** *p* < 0.01, *** *p* < 0.001 and **** *p* < 0.0001 using a two-tailed Student’s *t*-test or an ANOVA.

T cells play a crucial role in the adaptive immune response against cancer, allowing for the prognosis prediction of tumor patients and response to immune checkpoint blockade therapy [35,36]. Nevertheless, the mechanisms by which PHLDB2 regulates T cell function are still unclear. Based on the association of PHLDB2 with immunomodulator-related genes in HNSCC, we hypothesize that PHLDB2 may be involved in regulating the immune response to tumors, particularly in T cell activation. To confirm the functional role of PHLDB2 in T cell regulation, we conducted co-culture experiments involving T cells and HNSCC cells. Human T cells labeled with carboxyfluorescein diacetate succinimidyl ester (CFSE) were cultured with anti-CD3. A flow cytometry analysis revealed that the knockdown of PHLDB2 resulted in the increased growth and proliferation of T cells when co-cultured with PHLDB2 knockdown cells compared to control cells (Figure 5A). Furthermore, an examination of T cell activation markers demonstrated a significant increase in the frequencies of IL2^+^ T, IFNγ^+^ T, and GZMB^+^ T cells when cocultured with PHLDB2 knockdown cells (Figure 5B). To assess the T cell-mediated killing ability of tumor cells, we performed co-culture experiments using activated human peripheral blood T cells and HNSCC cells. The results demonstrated that PHLDB2 knockdown cells were more susceptible to CD3/CD28-activated human T cell-induced killing, as indicated by the elevated percentage of cleaved caspase-3^+^ HNSCC cells compared to the control group (Figure 5C,D). Accordingly, it can be concluded that a reduction in PHLDB2 in HNSCC cells has the capacity to regulate T cell function.

To further address the potential impact of PHLDB2 knockdown on T cells, murine HNSCC samples were harvested for a flow cytometry analysis. The results revealed that the depletion of PHLDB2 dramatically increased the percentage of CD8^+^ T cells as compared to the control groups (Figure 5E). However, there was no notable difference in the percentage of CD4^+^ T cells (Figure 5H). More importantly, the percentages of GZMB^+^CD8^+^ TILs and IFN-γ^+^CD8^+^ TILs (Figure 5F,G) and GZMB^+^CD4^+^ TILs and IFN-γ^+^CD4^+^ TILs (Figure 5I,J) showed significant increases in mice upon PHLDB2 knockdown.

These results suggest that targeting PHLDB2 promotes the activation and infiltration of T cells in HNSCC, which aligns with the conclusions drawn from the database analysis. Thus, targeting PHLDB2 may enhance the anti-tumor function of T cells and potentially exert anti-tumor immunity in HNSCC cells rather than directly suppressing tumor cell proliferation.

## 4. Discussions

The mounting evidence strongly supports the crucial role of PHLDB2 in tumorigenesis and the prognosis of cancer patients. The upregulation of PHLDB2 has been observed in various cancers, including colorectal cancer, gastric cancer, and lung adenocarcinoma [17,19,21,37]. However, the expression, function, and therapeutic significance of PHLDB2 in HNSCC remain insufficiently documented. In this study, we investigated the overexpression of PHLDB2 in HNSCC and its association with poor OS in HNSCC patients. According to previous research, PHLDB2 is involved in diverse cellular functions and signaling regulation. Yu et al. discovered that PHLDB2 acts as a significant oncogenic protein, able to bind to MDM2 and enhance the MDM2-mediated degradation of E-cadherin, thus promoting the EMT and metastasis [14]. Moreover, researchers have hypothesized that PHLDB2 could serve as a valuable biomarker and a potential target for interventions in colorectal cancer [38]. Notably, a recent report revealed that PHLDB2 plays a critical role in cetuximab resistance and is suggested as a potential therapeutic target for colorectal cancer [16]. Consistent with these findings, our study demonstrated that PHLDB2 promoted the EMT phenotype of cancer cells, facilitating malignant tumor progression in HNSCC. Silencing PHLDB2 significantly impeded cancer cell migration, while our in vitro experiments revealed no impact of PHLDB2 on cell proliferation in HNSCC cells. However, in vivo experiments showed that PHLDB2 markedly inhibited tumor growth. Recent studies have shown that tumor cells undergoing the EMT can construct an immunosuppressive tumor microenvironment by secreting certain cytokines or chemokines which, in turn, promote tumor development [25]. These findings provide further insights into the possible intrinsic mechanisms by which PHLDB2 exerts its antitumor effects by affecting the HNSCC tumor microenvironment.

To investigate the correlation between levels of PHLDB2 expression and the progression of HNSCC, we initially examined co-expressed genes in conjunction with PHLDB2 using gene expression data from HNSCC patients in the TCGA database. LinkedOmics conducted this analysis. The results displayed a close association between PHLDB2 and the regulation of T cell activation. Additionally, when PHLDB2 was silenced, it significantly increased the proliferation and activation of T cells in vitro. A significant finding of this study was that PHLDB2 modified tumor-infiltrated T cells to optimize antitumor immune responses in HNSCC. By analyzing the TCGA database, we observed that PHLDB2 was highly expressed and showed an inverse correlation with T cell infiltration in HNSCC. These discoveries indicate that PHLDB2 may promote immune escape in HNSCC by influencing the function of T cells. Consequently, blocking PHLDB2 could be a promising strategy for anti-tumor purposes. To further validate the impact of PHLDB2 on T cell function in HNSCC, an immunocompetent C3H HNSCC mouse model was established and treated with PHLDB2-transfected SCC7 cells or a control. The results showed that tumors with PHLDB2 knockdown significantly impede the growth of HNSCC by inducing CD8^+^ T cell-mediated anti-tumor immunity. Comparable outcomes were observed in T cell cytotoxicity assays in vitro. These results support the idea that the targeted inhibition of PHLDB2 improves CD8^+^ T cell-mediated antitumor immunity in HNSCC. However, the detailed molecular mechanism by which PHLDB2 depletion mediates the infiltration and cytotoxicity of CD8^+^T cells was not investigated in the present study. A growing body of evidence has shown that anti-PD-1 antibodies are a useful therapeutic strategy for the treatment of patients with refractory or metastatic HNSCC [39,40]. However, due to immune resistance, the objective response rate to anti-PD-1 treatment in HNSCC is extremely low. In order to improve the efficacy of tumor immunotherapy, ICB-based combination therapies have been developed and have demonstrated promising activity, including combinations of anti-PD1/PD-L1 with chemotherapy, other immune checkpoint inhibitors (such as CTLA4, LAG3, and TIM3), cetuximab, CAR-T, EZH2 inhibitors, TGF-β inhibitors, and cancer vaccines [41,42,43,44]. The main reasons for the unexpected therapeutic effect of immunotherapy in tumors are cytotoxic T cell infiltration, PD-1/PD-L1 expression, neo-antigen generation, and an immunosuppressive tumor microenvironment [45,46]. In this study, we show that PHLDB2 depletion induces effective antitumoral immunity through recruitment of cytotoxic CD8^+^ T cells and the remodeling of the tumor microenvironment in HNSCC, which suggests that targeting PHLDB2 may be an effective strategy to improve PD-1 immunotherapy. In our next study, we will further investigate whether the targeted inhibition of PHLDB2 in combination with a PD-1 monoclonal antibody improves clinical outcomes in patients with head and neck squamous cell carcinoma.

Our findings have shown that the expression of PHLDB2 acts as an autonomous prognostic indicator and potentially significantly contributes to the promotion of the EMT in HNSCC. Moreover, the removal of PHLDB2 leads to a notable increase in the activation and infiltration of CD8^+^ T cells in HNSCC.

## Figures and Tables

**Table 1:** Correlation between PHLDB2 expression and the clinicopathological features of the HNSCC cases from The Cancer Genome Atlas (TCGA).

Characteristic	Low Expression of PHLDB2	High Expression of PHLDB2	*p*-Value
*n*	251	251	
T stage, *n* (%)			0.024
T1	21 (4.3%)	12 (2.5%)	
T2	81 (16.6%)	63 (12.9%)	
T3	66 (13.6%)	65 (13.3%)	
T4	75 (15.4%)	104 (21.4%)	
N stage, *n* (%)			0.780
N0	120 (25%)	119 (24.8%)	
N1	40 (8.3%)	40 (8.3%)	
N2	77 (16%)	77 (16%)	
N3	2 (0.4%)	5 (1%)	
M stage, *n* (%)			0.216
M0	234 (49.1%)	238 (49.9%)	
M1	4 (0.8%)	1 (0.2%)	
Clinical stage, *n* (%)			0.524
Stage I	12 (2.5%)	7 (1.4%)	
Stage II	51 (10.5%)	44 (9%)	
Stage III	50 (10.2%)	52 (10.7%)	
Stage IV	131 (26.8%)	141 (28.9%)	
Gender, *n* (%)			0.034
Female	78 (15.5%)	56 (11.2%)	
Male	173 (34.5%)	195 (38.8%)	
Age, *n* (%)			0.395
≤60	117 (23.4%)	128 (25.5%)	
>60	133 (26.5%)	123 (24.6%)	
Histologic grade, *n* (%)			<0.001
G1	43 (8.9%)	19 (3.9%)	
G2	125 (25.9%)	175 (36.2%)	
G3	70 (14.5%)	49 (10.1%)	
G4	1 (0.2%)	1 (0.2%)	
Smoker, *n* (%)			0.015
No	67 (13.6%)	44 (8.9%)	
Yes	178 (36.2%)	203 (41.3%)	
Alcohol history, *n* (%)			0.106
No	89 (18.1%)	69 (14.1%)	
Yes	160 (32.6%)	173 (35.2%)	
Age, median (IQR)	61 (55, 69)	60 (53, 67)	0.187

## Data Availability

The data presented in this study are available upon request from the corresponding author.

## Supplementary Materials

The following supporting information can be downloaded at https://www.mdpi.com/article/10.3390/biom14020232/s1, Figure S1: Kaplan–Meier survival curve analysis of the prognostic significance of high and low levels of expression of PHLDB2 in human HNSCC using TCGA; Figure S2: Statistical chart of Western blot, wounding healing and transwell assays; Figure S3: Correlation analysis of PHLDB2 expression and immune infiltration in HNSCC; Figure S4: The correlation of PHLDB2 expression and chemokines and/or chemokine receptors. Original images of Figure 1E and Figure 2B.
