# Supplementary material for: Depletion of PHLDB2 Suppresses Epithelial–Mesenchymal Transition and Enhances Anti-Tumor Immunity in Head and Neck Squamous Cell Carcinoma
Source: Biomolecules. 2024 Feb 17;14(2):232. doi: 10.3390/biom14020232 (PMC10886581; doi:10.3390/biom14020232)
Supplement: Supplementary file 1 [file biomolecules-14-00232-s001.zip › biomolecules-2839057-supplementary.pdf]

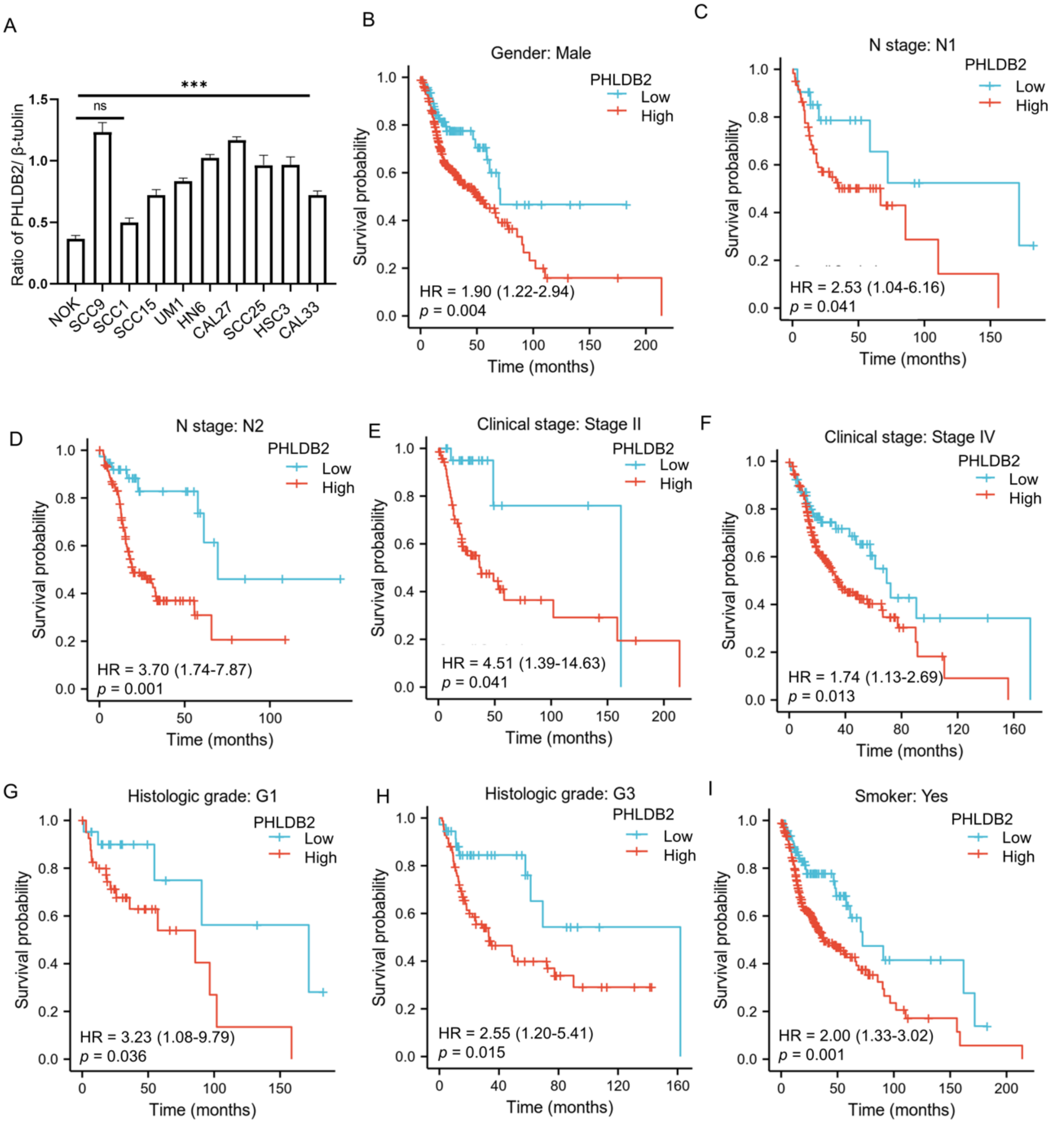

Figure. S1 Kaplan–Meier survival curve analysis of the prognostic significance of a high and a low expression of PHLDB2 in human HNSCC using TCGA.

(A) Statistical analysis of the immunoblotting data for EMT-related markers ,(B–H) Kaplan–Meier estimates of the overall survival probability of TCGA patients in all HNSCC patients. Subgroup analysis for male patients (B), N1 (C), N2 (D), clinical

staging stage II (E), clinical staging stage IV (F), histological grade G1 (G), and histological grade G3 (H) and smokers (I).

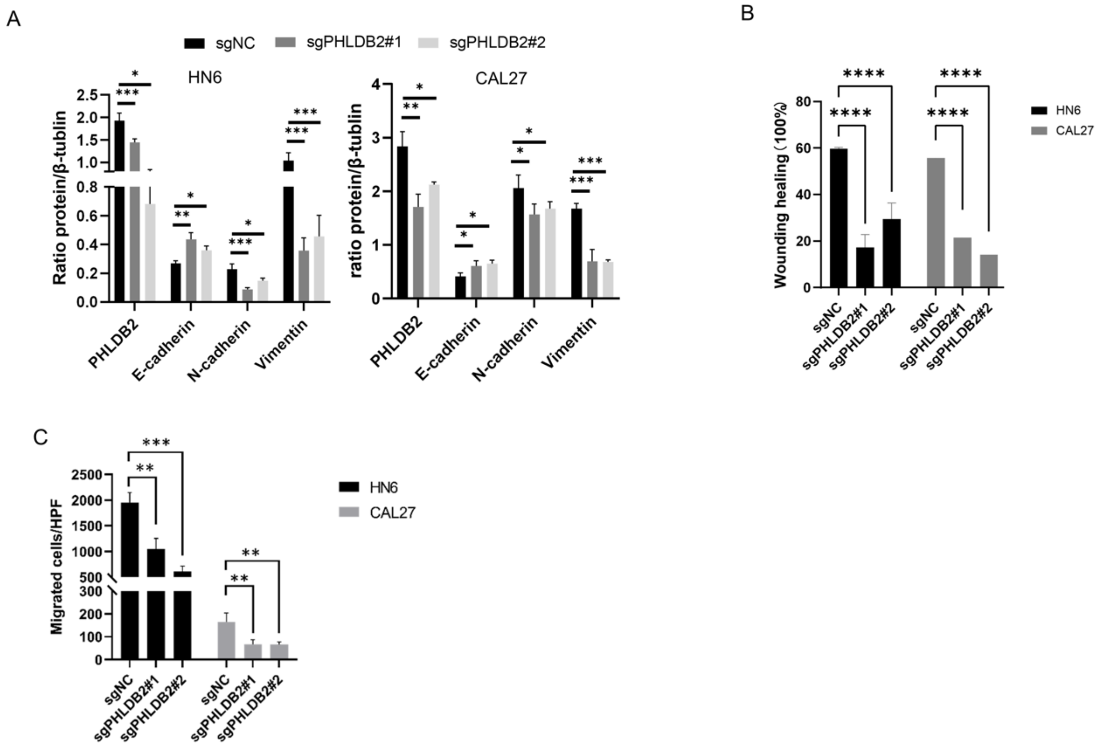

Figure. S2 Statistical analysis of the Weatern Blot, wound-healing and transwell assays of sgNC and sgPHLDB2 HNSCC cells.

(A) Statistical analysis of the Weatern Blot assays of PHLDB2 expression and the EMT related phenotype of sgNC and sgPHLDB2 HNSCC cells. (B) Statistical analysis of the wound-healing assays of sgNC and sgPHLDB2 HNSCC cells. (C) Statistical analysis of the transwell assays of sgNC and sgPHLDB2 HNSCC cells.

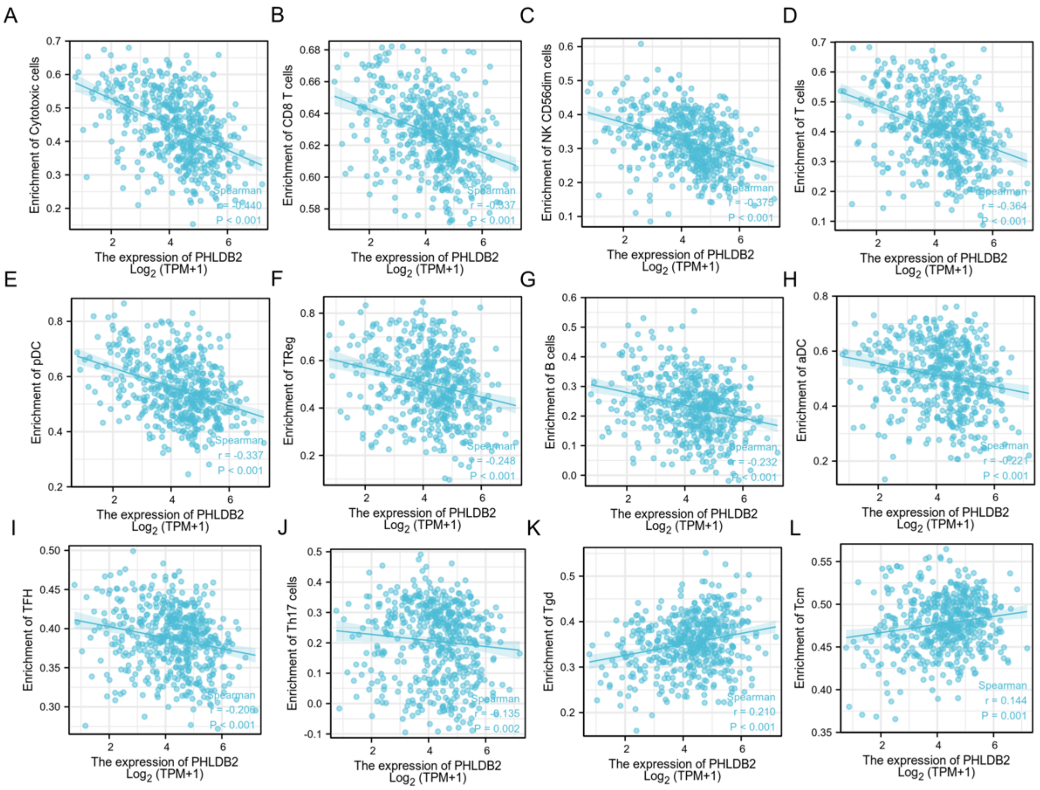

Figure. S3 Correlation analysis of PHLDB2 expression and immune infiltration in HNSCC.

Correlation between the expression level of PHLDB2 and immune infiltration in HNSCC: cytotoxic cells (A), CD8+ T cells (B), NK CD56bright cells (C), T cells (D), pDC (E), Treg (F), B cells (G), aDC (H), TFH (I), Th17 (J), Tgd (K), Tcm (L). *$p < 0.05$, **$p < 0.01$, ***$p < 0.001$.

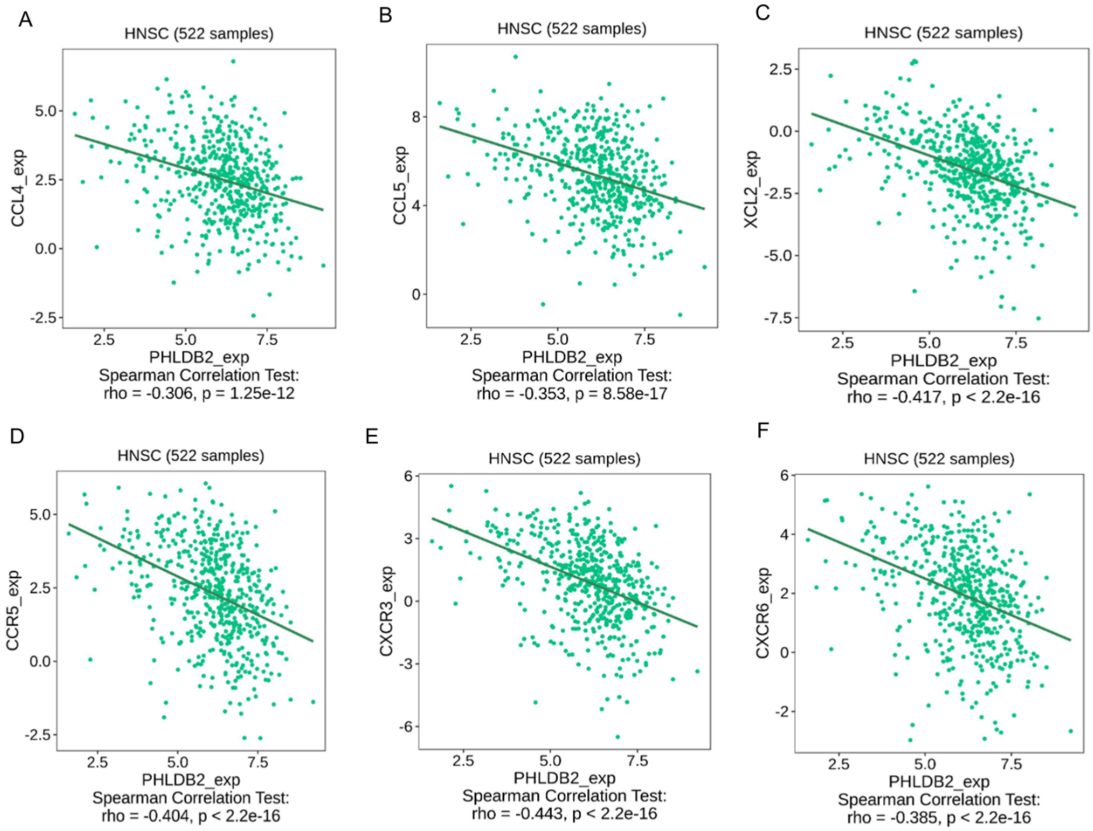

Figure. S4 The correlation of PHLDB2 expression and chemokines and/or chemokine receptors.

(A-F) PHLDB2 expression in HNSCC is negatively correlated with CCL4, CCL5, XCL2, CCR5, CXCR3, and CXCR6.
